# Fractional flow reserve of non-culprit vessel post-myocardial infarction: is it reliable?

**DOI:** 10.1186/s12872-015-0122-1

**Published:** 2015-10-14

**Authors:** Luís Leite, Joana Moura Ferreira, João Silva Marques, Elisabete Jorge, Vítor Matos, Jorge Guardado, João Calisto, Mariano Pego

**Affiliations:** Departament of Cardiology, Centro Hospitalar e Universitário de Coimbra, Praceta Prof. Mota Pinto, Coimbra, 3000-075 Portugal

**Keywords:** Multi-vessel disease, Myocardial infarction, Fractional flow reserve, Ischemia, Plaque vulnerability

## Abstract

**Background:**

Multi-vessel disease is frequent in patients presenting with myocardial infarction and have an important prognostic impact. The decision to proceed to revascularization in non-culprit vessels can be postponed until ischemia is proven in non-invasive stress tests. On the other hand, there is an increasing evidence to support the role of fractional flow reserve (FFR) in acute coronary syndrome setting.

**Case presentation:**

We report a case in which a FFR-guided strategy for non-culprit vessels, 3 weeks after an ST-segment elevation myocardial infarction, was followed by a short-term sub-occlusion of the evaluated vessel.

**Conclusion:**

The timing of the coronary microcirculation recovery post-myocardial infarction, avoiding a possible false negative FFR, and the diagnostic gaps between ischemia and plaque vulnerability are under discussion. An FFR-guided strategy in this setting should be interpreted with caution.

## Background

The prevalence of multi-vessel disease (MVD) in patients presenting with acute ST-segment elevation myocardial infarction (STEMI) approaches 40 % [1]. This subgroup of STEMI patients has a higher risk of major adverse cardiac events (MACE) in the first year after primary percutaneous coronary intervention (PCI) [2]. Therefore, the assessment of the actual severity of the non-culprit coronary artery stenosis and its optimal treatment is clinically important soon after primary PCI.

## Case presentation

**Fig. 1:**
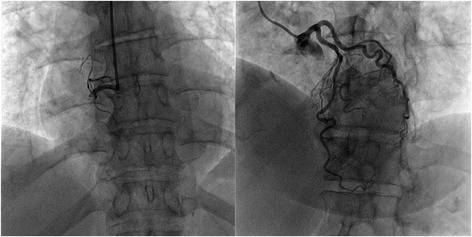
Coronary angiography in the inferior STEMI. Total occlusion of the proximal-segment of the right coronary artery and three intermediate stenosis in the mid-segment of the LAD

A 79-year-old female with a history of hypertension and dyslipidaemia, was admitted for an inferior STEMI and underwent primary angioplasty of the right coronary artery with implantation of a 3.0 × 33 mm Xience™ everolimus-eluting stent. The emergency coronary angiography also showed three intermediate stenosis in the mid-segment of the left anterior descending artery (LAD) – Fig. 1. Transthoracic echocardiogram demonstrated normal biventricular systolic function with a left ventricular ejection fraction of 60 %. The patient was clinically stable and was discharged 4 days later with optimized medical therapy (aspirin 100 mg qd, ticagrelor 90 mg bid, atorvastatin 40 mg qd, carvedilol 6.25 mg bid, ramipril 1.25 mg qd, pantoprazol 40 mg qd).

**Fig. 2:**
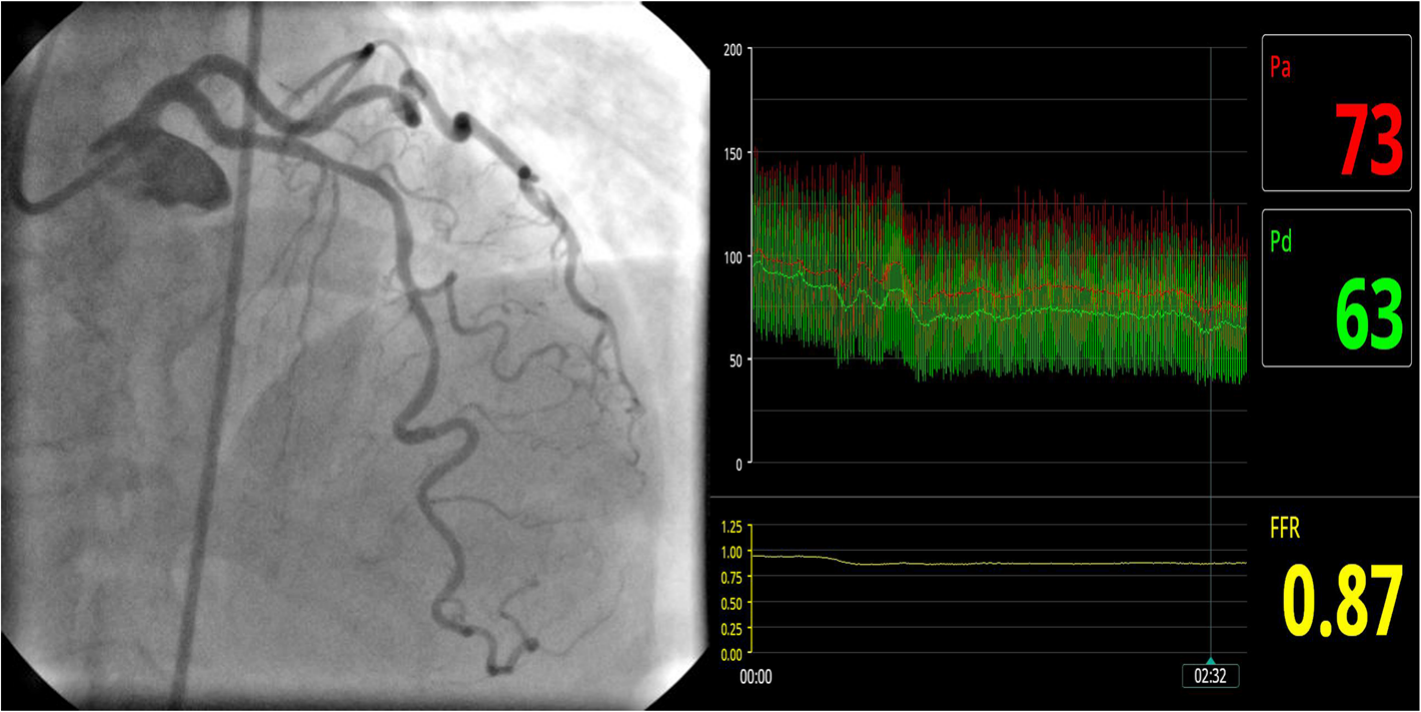
Three-week follow-up with coronary angiography and FFR. Coronary angiography showing the intermediate LAD lesions and FFR evaluation with the result of 0.87

In order to assess the hemodynamic relevance of the LAD lesions, a 3 week scheduled coronary angiography with fractional flow reserve (FFR) measurements of LAD was performed (Fig. 2), using a 0.014″ pressure guide wire (PressureWire Aeris™, St Jude Medical, Uppsala, Sweden). Resting distal coronary pressure to aortic pressure ratio (Pd/Pa) was 0.94. FFR was 0.87, indicating physiologically non-significant stenosis. There was no damping of the proximal aortic pressure trace, ensuring an accurate FFR measurement.

**Fig. 3:**
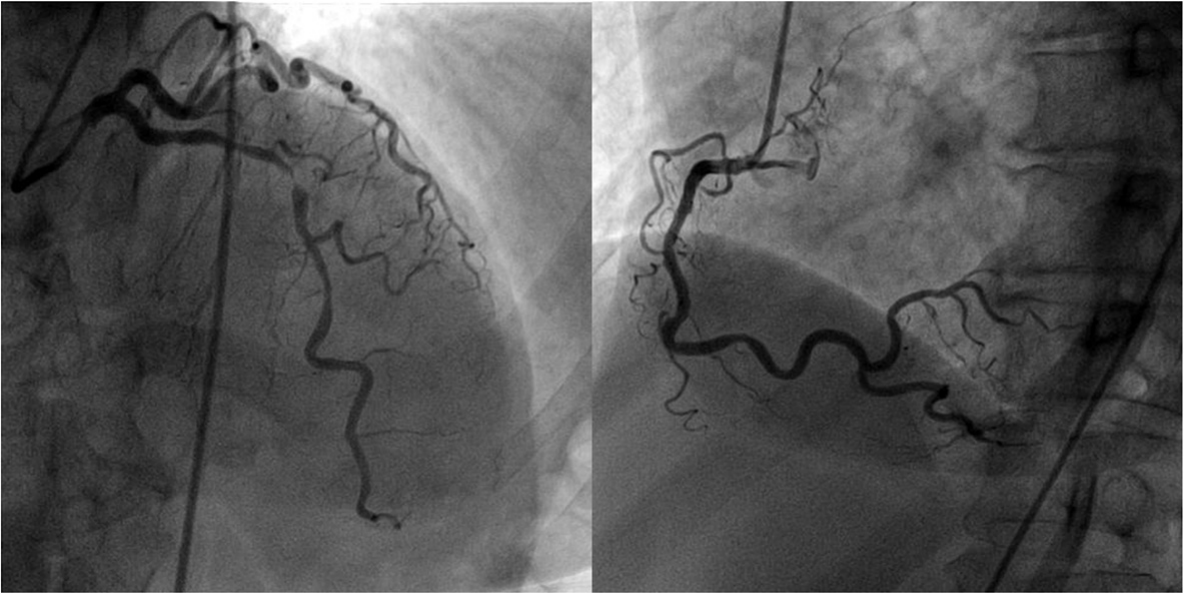
Coronary angiography in the anterior STEMI. Sub-occlusion of the mid-LAD and right coronary artery without significant lesions

Two months later, the patient was admitted for an anterior STEMI and the emergency coronary angiography revealed a sub-occlusion of the mid-LAD (Fig. 3). The lesion was treated with a 3.0 × 15 mm Xience™ everolimus-eluting stent. Right coronary angiography showed neither restenosis in the previously implanted stent nor other significant coronary lesions. The patient assured having good compliance with the therapeutic regimen since the first cardiovascular event.

## Discussion

In patients with STEMI and MVD, the current recommendations state that primary PCI should be limited to the culprit vessel with the exception of cardiogenic shock in the presence of multiple, critical stenosis or highly unstable lesions, and if there is persistent ischemia after PCI on the supposed culprit lesion [3]. If staged PCI to non-culprit vessels is being considered, non-invasive stress testing (myocardial perfusion scintigraphy, stress echocardiography, positron emission tomography or magnetic resonance imaging) should be used for ischemia and viability testing prior to a decision to proceed with PCI [4].

Fractional flow reserve is a well-validated technique to guide coronary intervention by identification of lesion-level ischemia. In the DEFER study, the prognosis of intermediate lesions with an FFR of > 0.75 was excellent if treated medically, with a < 1 % risk of any AMI after 5 years of follow-up [5]. However, studies to date have mainly involved stable patients outside of acute myocardial infarction (AMI) [5–8]. The use of FFR to assess culprit and non-culprit lesions in the setting of acute ischemia represents a controversial area, although there is an increasing evidence base to support the role of FFR-guided strategy in acute coronary syndrome (ACS) [9].

The physiological principles underlying FFR are critically dependent on the ability to achieve maximal hyperaemia. In patients with AMI, pathophysiological disturbances in the microvasculature can have a potential impact on the ability to induce maximal hyperaemia, and thereby compromise the accuracy of FFR assessments in non-culprit vessels, theoretically leading to false negative results [10]. The 2011 ESC Guidelines for the management of ACS without ST-segment elevation [11] recommend that FFR should be ideally performed more than 5 days after the acute event in order to minimize the impact of any microvascular disturbance. The 2012 ESC Guidelines for the management of STEMI [4] state the staged revascularization approach for STEMI with MVD can be guided by FFR if performed several days or weeks after primary PCI. In the case report presented, the functional assessment of the mid-LAD lesions was made 3 weeks later than the primary PCI, in concordance to the current guidelines.

One of the most important studies about the use of FFR in ACS was from Ntalianis et al. [12] who studied 75 acute STEMI patients and 26 NSTEMI patients (<72 h post onset) and measured FFR in the non-culprit stenosis immediately following PCI of the culprit vessel and then repeated the FFR at 35 ± 4 days post initial procedure. The FFR value remained unchanged between the acute and follow-up phases in patients with STEMI (0.78 ± 0.10 vs. 0.76 ± 0.10, *p* = NS) and NSTEMI (0.77 ± 0.10 vs. 0.77 ± 0.20, *p* = NS). In only 2 patients, the FFR value was higher than 0.80 at the acute phase and lower than 0.75 at follow-up. The authors stated the data support that FFR measurements are safe and reliable for evaluating the severity of non-culprit stenosis in the acute phase of ACS, even during primary PCI.

A more recent study from Cuculi et al. [13] performed an invasive assessment of coronary physiology in 82 STEMI patients, immediately following PCI of the culprit vessel and 6 months later, associated with a contrast-enhanced cardiac magnetic resonance imaging evaluation of microvascular obstruction. Baseline Pd/Pa remained stable over time, but FFR reduced significantly between primary PCI and 6 months (*p* = 0.008); this reduction was mainly observed in patients with microvascular obstruction. Therefore, the authors stated that coronary microcirculation recovery progresses further by 6 months, suggesting that using FFR soon after STEMI might underestimate the degree of non-culprit vessel stenosis in almost half of patients.

The index of microcirculatory resistance (IMR) is a validated invasive wire-based measure of microvasculature function [14] and could be useful to assess the status of the coronary microcirculation in the setting of AMI [15], which may be used to validate an FFR result. The other potential caveat in a FFR-guided strategy in ACS patients, as it was done in our case report, is the possible existence of vulnerable coronary plaques in absence of flow limitation [9]. In the Providing Regional Observations to Study Predictors of Events in the Coronary Tree (PROSPECT) study [16], patients presenting with ACS in whom PCI was successful, underwent 3-vessel radiofrequency intravascular ultra-sound (IVUS) imaging, and were followed for a median of 3.4 years for the incidence of MACE. In this study, the non-culprit lesions that led to MACE were frequently mild on angiographic assessment, but most were characterized by a large plaque burden, a small luminal area and were thin-cap fibroatheromas (TCFA); no MACE arose from untreated segments with a plaque burden resulting in less than 40 % loss of cross-sectional luminal area. Using data from PROSPECT study, some authors stated that clinical and angiographic characteristics had poor predictive accuracy in identifying patients with untreated high-risk plaques, at least not enough to obviate the need for intracoronary-imaging, although they assume that it is unrealistic to applicate 3-vessel invasive imaging in every clinical setting [17]. Others authors divided the same cohort into quartiles according to baseline angiographic diameter stenosis and concluded that the triad of predictors of future MACE increased in frequency with increasing angiographic diameter stenosis [18].

Some points of controversy involve the risk assessment of future MACE: do we have a cross-link between ischemia and plaque vulnerability? Can we expect that plaques with higher FFR values are stable and that lower FFR, with repetitive ischemia and high shear stress, induce vulnerability? Or do we have a diagnostic gap of vulnerable plaque between physiology and morphology? A study from Versteeg et al. [19] demonstrated that monocyte toll-like receptors 2 and 4 related to plaque vulnerability were significantly higher in patients with FFR < 0.75 than in patients with an FFR measurement of > 0.80, suggesting that vulnerability may be preceded by ischemia. In the Fractional Flow Reserve and Intravascular Ultrasound Relationship Study (FIRST) [20], the aim was to evaluate the correlation between FFR, IVUS, and virtual histology (VH) in intermediate coronary lesions. Minimal lumen area obtained by IVUS showed a moderate correlation with FFR measurements and the optimal cut-off for a FFR of < 0.80 varied depending on the vessel size. Plaque composition assessed by VH-IVUS identified only plaque burden as having any correlation with FFR values. Lesions without TCFA had better correlation with FFR compared with lesions with TCFA. A study from Hüseyinova et al. [21] evaluated 48 non-ST-elevation ACS patients having paired hemodynamic and morphological data of the culprit vessel. It was demonstrated that for a given stenosis, FFR values decrease with an increase in necrotic core and dense calcium contents of the physiologically significant coronary plaques. However, plaque composition did not exert any influence on the hemodynamic effect generated by physiologically non-significant stenosis.

The COMPARE ACUTE trial is an ongoing study enrolling MVD patients undergoing primary PCI and randomly allocates patients to receive either FFR-guided PCI or culprit vessel-only PCI in the setting of STEMI [22]. This trial may help to define the role of FFR in STEMI patients with MVD.

## Conclusions

The use of FFR to assess non-culprit lesions in AMI patients is useful to guide treatment if strongly indicative of ischemia. A negative FFR is this setting should be interpreted with caution and may be appropriate to access plaque vulnerability by intracoronary imaging, to consider subsequent non-invasive testing or alternatively repeat FFR at a later date. Further studies are required to stablish the ideal timing to perform FFR after AMI, in order to minimize the impact of any microvascular disturbance. The role of FFR in the assessment of vulnerable plaques *per se* also requires additional evaluation in clinical trials.

### Consent

Written informed consent was obtained from the patient for publication of this case report and any accompanying images. A copy of the written consent is available for review by the Editor of this journal.

## Abbreviations

ACS: Acute coronary syndrome
AMI: Acute myocardial infarction
FFR: Fractional flow reserve
IMR: Index of microcirculatory resistance
IVUS: Intravascular ultra-sound
LAD: Left anterior descending artery
MACE: Major adverse cardiac events
MVD: Multi-vessel disease
PCI: Percutaneous coronary intervention
Pd/Pa: Distal coronary pressure to aortic pressure ratio
STEMI: ST-segment elevation myocardial infarction
TCFA: Thin-cap fibroatheromas
VH: Virtual histology
